# Adjuvant immunotherapy for esophageal squamous cell carcinoma after neoadjuvant chemoimmunotherapy: a multicenter real-world study

**DOI:** 10.1097/JS9.0000000000003546

**Published:** 2025-09-24

**Authors:** Chunji Chen, Ziqiang Tian, Jiangbo Lin, Xuefeng Leng, Jianfei Shen, Jinbo Zhao, Huilai Lv, Changchun Wang, Xinyu Mei, Yongtao Han, Qifeng Wang, Jiahua Lv, Hainan Chen, Xiaolong Yan, Zhichao Liu, Zhengyang Zhang, Qihong Zhong, Youhua Jiang, Liwei Xu, Xiaoyang Li, Dong Qian, Dehua Ma, Minhua Ye, Chunguo Wang, Zimin Wang, Zhigang Li, Xufeng Guo

**Affiliations:** a Department of Thoracic Surgery, Shanghai Chest Hospital, Shanghai Jiao Tong University School of Medicine, Shanghai, China; b Department of Thoracic Surgery, The Fourth Hospital of Hebei Medical University, Shijiazhuang, China; c Clinical Research Center for Thoracic Tumors of Fujian Province, Fuzhou, China; d Department of Thoracic Surgery, Fujian Medical University Union Hospital, Fuzhou, China; e Key Laboratory of Cardio-Thoracic Surgery (Fujian Medical University), Fujian Province University, Fuzhou, China; f Department of Thoracic Surgery, Sichuan Cancer Hospital and Research Institute, School of Medicine, University of Electronic Science and Technology of China (UESTC), Chengdu, China; g Department of Thoracic Surgery, Taizhou Hospital of Zhejiang Province affiliated to Wenzhou Medical University, Linhai, China; h Key Laboratory of Minimally Invasive Techniques & Rapid Rehabilitation of Digestive System Tumor of Zhejiang Province, Linhai, China; i Department of Thoracic Surgery, Tangdu Hospital, Air Force Medical University, Xi’an, China; j Department of Thoracic Surgery, Zhejiang Cancer Hospital, Hangzhou, China; k Department of Thoracic Surgery, The First Affiliated Hospital of USTC, University of Science and Technology of China (USTC), Hefei, China; l Division of Life Sciences and Medicine, University of Science and Technology of China (USTC), Hefei, China; m Department of Radiation Oncology, Sichuan Cancer Hospital and Research Institute, School of Medicine, University of Electronic Science and Technology of China (UESTC), Chengdu, China; n Department of Radiation Oncology, The First Affiliated Hospital of USTC, University of Science and Technology of China (USTC), Hefei, China; o EnzeHospital, Taizhou Enze Medical Center (Group), Taizhou, China

**Keywords:** adjuvant immunotherapy, esophageal squamous cell carcinoma, multicenter real-world study, neoadjuvant chemoimmunotherapy

## Abstract

**Background:**

Although adjuvant immunotherapy demonstrated an improvement in disease-free survival (DFS) within the chemoradiotherapy cohort of the CheckMate 577 trial, its efficacy and role following NCIT remain to be elucidated. This large-sample, multicenter, real-world study aims to assess survival benefits of adjuvant immunotherapy in esophageal squamous cell carcinoma (ESCC) patients treated with neoadjuvant chemoimmunotherapy (NCIT) followed by R0 resection.

**Methods:**

This multicenter retrospective study (eight centers, January 2019–March 2023) included 724 ESCC patients undergoing NCIT and R0 resection. Propensity score matching (PSM) balanced 262 patients per group: NCIT + Surgery (NCIT + S) vs. NCIT + Surgery + adjuvant immunotherapy [NCIT + S + ICI (immune checkpoint inhibitors)]. Primary endpoints were 2-year overall survival (OS) and DFS; secondary endpoints included recurrence patterns.

**Findings:**

Median follow-up: 31.2 months (IQR 24.0–39.9). Post-PSM analysis showed no significant OS benefit [2-year OS: 80.0% vs. 84.5%, hazard ratio (HR) = 1.15, 95% confidence interval (CI): 0.78–1.70, *P* = 0.12] or DFS improvement (77.7% vs. 72.6%, HR = 0.97, 95% CI: 0.69–1.37, *P* = 0.69) for NCIT + S vs. NCIT + S + ICI. Adjuvant immunotherapy was not independently protective for OS (HR = 0.87, *P* = 0.48) or DFS (HR = 1.03, *P* = 0.87). However, subgroup analyses revealed OS benefits in Non-MPR (major pathological response) patients (63.9% vs. 81.7%, HR = 1.78, 95% CI: 1.05–3.03, *P* = 0.035) and non- pathological complete response (pCR) patients (74.9% vs. 84.3%, HR = 1.39, 95% CI: 1.19–2.10, *P* = 0.031). Recurrence rates did not differ (local: 15.3% vs. 20.2%, *P* = 0.50; distant: 16.8% vs. 21.6%, *P* = 0.17).

**Interpretation:**

Adjuvant immunotherapy provided no survival benefit in the overall NCIT-treated ESCC cohort but improved OS in patients with residual tumor (Non-MPR/Non-pCR). Further studies are warranted to refine patient selection for adjuvant immunotherapy in this setting.

**HIGHLIGHTS**

- **No overall survival benefit**: Adjuvant immunotherapy provided no significant improvement in 2-year overall survival [OS: 80.0% vs. 84.5%, hazard ratio (HR) = 1.15, *P* = 0.12] or disease-free survival (DFS: 77.7% vs. 72.6%, HR = 0.97, *P* = 0.69) for the overall cohort of esophageal squamous cell carcinoma (ESCC) patients treated with neoadjuvant chemoimmunotherapy (NCIT) followed by surgery.
- **Benefit in patients with residual disease**: Subgroup analyses revealed significant overall survival benefits from adjuvant immunotherapy specifically in patients who did not achieve a major pathological response (MPR) (Non-MPR: 81.7% vs. 63.9%, HR = 1.78, *P* = 0.035) or a pathological complete response (Non-pCR: 84.3% vs. 74.9%, HR = 1.39, *P* = 0.031).
- **Pathological response guides benefit**: The survival benefit of adjuvant immunotherapy was linked to the extent of residual tumor after NCIT, showing significant OS improvement in patients with the poorest response (TRG4: >50% residual tumor) and potential DFS benefit in patients with ypN1 disease, but no benefit in patients achieving MPR or pCR. Recurrence patterns were not altered.

## Introduction

Esophageal cancer ranks as the sixth most common cause of cancer death globally[1]. Nearly half of new cases worldwide are diagnosed in China every year, where the histological type is mainly esophageal squamous cell carcinoma (ESCC)[2]. Neoadjuvant chemoradiotherapy (NCRT) followed by surgery is a widely used standard of care for patients with resectable, locally advanced esophageal cancer^[3–6]^. However, the risk of recurrence after NCRT and surgery remains high, especially among the 70–75% of patients who do not have a pathological complete response (pCR)^[7,8]^. The majority of patients who received NCRT had a recurrence and metastasis rate of 34.7% during a median follow-up of 2 years after surgery, with a distant metastasis (DM) rate of 22% in the CROSS study[7]. Long-term follow-up results of the NEOCRTEC5010 study also showed that 33.0% of patients who had received NCRT still had recurrence and metastasis during the 3-year follow-up after surgery, and DM (21.4%) was more common[9]. All these evidence suggests that more efficient systemic treatment options are required to further improve efficacy in locally advanced resectable ESCC.

In recent years, several clinical trials of neoadjuvant chemoimmunotherapy (NCIT) have reported favorable pCR rate ranges from 16.7% to 55.6% with acceptable treatment-related toxicity, which has been the most promising research frontier for multidisciplinary treatment of locally advanced ESCC^[10–12]^. There were small sample retrospective studies suggested that NCIT may offer comparable or even better long-term survival outcomes than NCRT^[13–15]^. In addition, we have published a large scale real-world study, the results suggested that NCIT could offer higher 2-year OS and disease-free survival (DFS) compared to NCRT in patients with locally advanced ESCC[16], and it is expected to become an alternative treatment for NCRT in the future.

While the CheckMate577 trial has demonstrated that adjuvant immunotherapy significantly improve DFS in patients with locally advanced ESCC who underwent NCRT[17],with a median DFS of 22.4 months for those treated with nivolumab adjuvant therapy compared to 11.0 months for those receiving placebo [hazard ratio (HR) 0.69, 95% confidence interval (CI) 0.56–0.86, *P* < 0.001], the role of adjuvant immunotherapy in improving survival outcomes for patients who receive NCIT followed by surgery remains unclear, although relevant randomized clinical trials (RCTs) are ongoing^[18,19]^. Here, we present a comprehensive analysis of multicenter, real-world data collected from eight high-volume esophageal surgery centers in China, focusing on patients with locally advanced ESCC who received NCIT. This study aimed to provide important insights into the adjuvant immunotherapy after NCIT for locally advanced ESCC.

## Methods

The study has been reported in line with the STROCSS criteria[20]. Data were obtained from eight high volume esophageal surgery centers across China. As this study is retrospective, informed consent was not required.

### Participants

**Figure 1.**
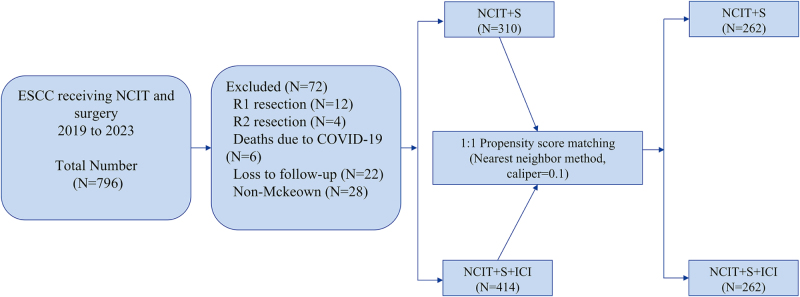
Trial profile.

We integrated data from 796 patients with locally advanced ESCC who received NCIT at eight high-volume esophageal surgery centers between 1 January 2019 and 31 March 2023. The inclusion criteria were: (1) clinical staging of ESCC prior to treatment, according to the 8th edition of the American Joint Committee on Cancer (AJCC) TNM staging system[21], with clinical tumor stage T1b, lymph node stage N1–3, metastasis stage M0, T2–4a, N0–3, M0; (2) age between 18 and 75 years; (3) normal hematologic, renal, hepatic, cardiac, and pulmonary function. The exclusion criteria included: (1) concomitant advanced malignancies; (2) patients with R1 or R2 resection; (3) patients who died due to COVID-19; (4) patients lost to follow-up. The patient screening process is illustrated in Figure 1.

### Procedures

The NCIT regimen comprises two cycles of immune checkpoint inhibitors (ICIs), such as pembrolizumab, nivolumab, tislelizumab, camrelizumab, sintilimab, or toripalimab, combined with chemotherapy consisting of a platinum agent (cisplatin, carboplatin, or nedaplatin) plus paclitaxel or fluorouracil. Adjuvant immunotherapy might be recommended for patients for 1 year after surgery following multidisciplinary discussions, basing on the pathological tumor stage and the patient’s recovery condition. The initial dose is typically administered 4–8 weeks post-surgery. All patients of the NCIT + S + ICI group had received at least one cycle of adjuvant immunotherapy.

Esophagectomy was generally scheduled within 4–6 weeks after completing NCIT. Patients underwent McKeown minimally invasive esophagectomy including thoraco-abdominal two-field lymphadenectomy (for middle/distal third tumors) or cervical-thoraco-abdominal three-field lymphadenectomy (for proximal third tumor). Dissection of bilateral recurrent laryngeal nerve nodes was mandatory[22].

### Pathological examination, follow-up, and outcomes

Pathological staging was performed according to the AJCC 8th edition TNM classification system. Each patient’s pathological samples were independently reviewed by two experienced pathologists at each center using the College of American Pathologists cancer protocol for esophageal carcinoma. R0 resection was defined as a radical excision with negative margins at the distal, proximal, and circumferential edges. pCR was defined as the absence of viable tumor cells at both the primary tumor site and in all resected lymph nodes. Tumor response to NCIT was assessed using the Chirieac tumor regression grade (TRG) system which classifies response into four categories[23]: TRG 1 (no residual tumor), TRG 2 (<10% residual tumor), TRG 3 (10–50% residual tumor), and TRG 4 (>50% residual tumor). TRG1 and TRG2 are defined as major pathological responses (MPRs), with less than 10% residual primary tumor, regardless of the lymph node status.

After surgery, patients underwent assessments every 3 months for the first 2 years, followed by every 6 months for the next three years, and annually thereafter. Survival status, disease progression, and additional treatments were documented at each follow-up, which included a combination of outpatient visit records, hospitalization logs, and telephone interviews. Routine evaluations comprised a comprehensive physical examination, upper gastrointestinal X-rays, cervical ultrasound, and thoracic combined with abdominal contrast-enhanced CT scans and/or MRI. When clinically indicated, PET/CT and endoscopic ultrasound-guided biopsies were also performed. In this study, the primary endpoints were 2-year OS and DFS. OS was measured from the start of treatment to death from any cause or the last follow-up. DFS was defined as the time from surgery to tumor recurrence, death, or the last follow-up. Locoregional recurrence (LR) was defined as recurrence in the regional lymph nodes and/or at the anastomosis site, while DM included metastasis to various organs as well as to the supraclavicular lymph nodes.

### Statistical analysis

We used nearest-neighbor matching to pair patients of NCIT + Surgery (NCIT + S) group with NCIT + Surgery + adjuvant immunotherapy (NCIT + S + ICI) group in a 1:1, non-replacement manner, with a caliper value set at 0.1. The balance of covariates before and after matching was assessed, and the standardized mean difference (SMD) was used to evaluate the balance of baseline characteristics between the two groups after propensity score matching (PSM). An SMD of less than 0.1 indicates good balance between the groups. Survival outcomes between the two groups were compared using Kaplan–Meier curves and the log-rank test. Cox proportional hazards regression was employed to assess prognostic factors for OS and DFS. Data were analyzed with R software (version 4.2, The R Foundation, Vienna, Austria) and SPSS (version 24.0). A two-sided *P*-value of less than 0.05 was considered statistically significant.

## Results

### Patient characteristics

A total of 724 patients were included in the study, with 310 patients in the NCIT + S group and 414 patients in the NCIT + S + ICI group. Prior to PSM, there were significant differences between the two groups in terms of age, smoking history, tumor location, clinical T category, and ECOG performance status (all *P* < 0.05). After 1:1 PSM, baseline differences were effectively balanced, with 262 patients remaining in each group. A comparison of baseline characteristics before and after matching is provided in Table 1. SMDs less than 0.10 indicated good balance in baseline characteristics between the groups (Supplemental Digital Content Figure S1, available at, http://links.lww.com/JS9/F201).

**Table 1:** Patient characteristics before and after propensity score matching

Variables	Before PSM						After PSM			
	Total (*n* = 724)	NCIT + S (*n* = 310)	NCIT + S + ICI (*n* = 414)	*P*	SMD		Total (*n* = 524)	NCIT + S (*n* = 262)	NCIT + S + ICI (*n* = 262)	*P*	SMD
Age, *n* (%)				**<0.05**						0.700	
<60	249 (34.4)	92 (29.7)	157 (37.9)		0.170		152 (29.0)	78 (29.8)	74 (28.2)		−0.034
≥60	475 (65.6)	218 (70.3)	257 (62.1)		−0.170		372 (71.0)	184 (70.2)	188 (71.8)		0.034
Gender, *n* (%)				0.050						0.457	
Male	578 (79.8)	237 (76.5)	341 (82.4)		0.155		411 (78.4)	202 (77.1)	209 (79.8)		0.067
Female	146 (20.2)	73 (23.5)	73 (17.6)		−0.155		113 (21.6)	60 (22.9)	53 (20.2)		−0.067
BMI, *n* (%)				0.077						0.928	
<18	34 (4.7)	15 (4.8)	19 (4.6)		−0.012		25 (4.8)	13 (5.0)	12 (4.6)		−0.018
18–24	440 (60.8)	174 (56.1)	266 (64.2)		0.169		297 (56.7)	150 (57.2)	147 (56.1)		−0.023
>24	250 (34.5)	121 (39.1)	129 (31.2)		−0.170		202 (38.5)	99 (37.8)	103 (39.3)		0.031
Smoking history, *n* (%)				**<0.05**						0.855	
No	249 (34.4)	121 (39.0)	128 (30.9)		−0.176		184 (35.1)	93 (35.5)	91 (34.7)		−0.016
Yes	475 (65.6)	189 (61.0)	286 (69.1)		0.176		340 (64.9)	169 (64.5)	171 (65.3)		0.016
Alcohol abuse, *n* (%)				0.147						0.701	
No	213 (29.4)	100 (32.3)	113 (27.3)		−0.111		154 (29.4)	79 (30.1)	75 (28.6)		−0.034
Yes	511 (70.6)	210 (67.7)	301 (72.7)		0.111		370 (70.6)	183 (69.9)	187 (71.4)		0.034
Tumor location, *n* (%)				**<0.05**						0.956	
Proximal third	90 (12.4)	30 (9.7)	60 (14.5)		0.137		62 (11.8)	30 (11.4)	32 (12.2)		0.023
Middle third	429 (59.3)	159 (51.3)	270 (65.2)		0.292		306 (58.4)	153 (58.4)	153 (58.4)		0.000
Distal third	205 (28.3)	121 (39.0)	84 (20.3)		−0.466		156 (29.8)	79 (30.2)	77 (29.4)		−0.017
Clinical T category, *n* (%)				**<0.05**						0.415	
cT1	11 (1.5)	4 (1.3)	7 (1.7)		0.031		7 (1.3)	2 (0.8)	5 (1.9)		0.084
cT2	64 (8.8)	21 (6.8)	43 (10.4)		0.118		42 (8.0)	20 (7.6)	22 (8.4)		0.028
cT3	556 (76.8)	259 (83.5)	297 (71.7)		−0.262		435 (83.0)	216 (82.4)	219 (83.6)		0.031
cT4a	93 (12.9)	26 (8.4)	67 (16.2)		0.212		40 (7.7)	24 (9.2)	16 (6.1)		−0.128
Clinical N category, *n* (%)				0.138						0.794	
cN0	108 (14.9)	50 (16.1)	58 (14.0)		−0.061		79 (15.1)	38 (14.5)	41 (15.6)		0.032
cN1	380 (52.5)	161 (52.0)	219 (52.9)		0.019		272 (51.9)	136 (51.9)	136 (51.9)		0.000
cN2	212 (29.3)	94 (30.3)	118 (28.5)		−0.040		160 (30.5)	83 (31.7)	77 (29.4)		−0.050
cN3	24 (3.3)	5 (1.6)	19 (4.6)		0.142		13 (2.5)	5 (1.9)	8 (3.1)		0.067
ECOG PS, *n* (%)				**<0.001**						0.662	
0	355 (49.0)	130 (41.9)	225 (54.4)		0.249		247 (47.1)	121 (46.2)	126 (48.1)		0.038
1	369 (51.0)	180 (58.1)	189 (45.6)		−0.249		277 (52.2)	141 (53.8)	136 (51.9)		−0.038

BMI, body mass index, ECOG-PS, Eastern Cooperative Oncology Group Performance Status; ICI, immune checkpoint inhibitor; NCIT, neoadjuvant chemoimmunotherapy; PSM, propensity score matching; S, surgery; SMD, standardized mean difference.

The bold values represent *P* < 0.05.

### Pathological examination

Comparison of pathological results between the two groups showed that a higher Non-MPR proportion of patients in the NCIT + S + ICI group (43.9% vs. 34.7%, *P* = 0.032) and also higher Non-pCR proportion (84.4% vs. 71.0%, *P* < 0.001) than those in the NCIT + S group. Additionally, patients in the NCIT + S + ICI group had a later pathological stage compared to the NCIT + S group (ypN +: 50.8% vs. 38.9%, *P* = 0.045; AJCC p-staging III + IV: 51.9% vs. 39.3%, *P* = 0.004). No significant differences were observed between the two groups in terms of ypT category (Supplemental Digital Content Table S1, available at, http://links.lww.com/JS9/F202).

### Postoperative complications

Overall complications occurred in 44.1% (231/524) of the cohort, with a numerically higher incidence in the NCIT + S + ICI group (47.3% vs. 40.8%, *P* = 0.13). Respiratory complications were observed in 31.7% (166/524) of patients. Notably, pneumonia rates were significantly lower in the NCIT + S + ICI group (22.1% vs. 29.8%, *P* = 0.05), while respiratory failure (2.9% overall) and Acute Respiratory Distress Syndrome(ARDS) (1.3% overall) did not differ between groups (*P* = 0.43 and *P* = 1.00, respectively). The requirement for effusion treatment showed a trend toward reduction with ICI (21.8% vs. 28.2%, *P* = 0.09) (Supplemental Digital Content Table S2, available at, http://links.lww.com/JS9/F202).

### Survival

**Figure 2.**
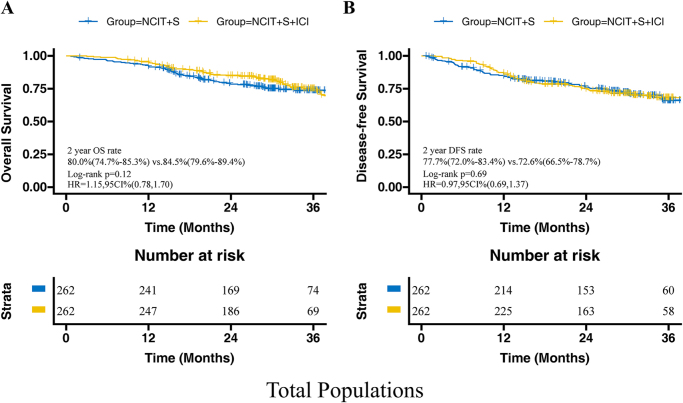
Kaplan–Meier curves of overall survival (A) and disease-free survival (B) between NCIT + S and NCIT + S + ICI group in total populations. ESCC, esophageal squamous cell carcinoma; NCIT, neoadjuvant chemoimmunotherapy; S, surgery; ICI, immune checkpoint inhibitor.

**Figure 3.**
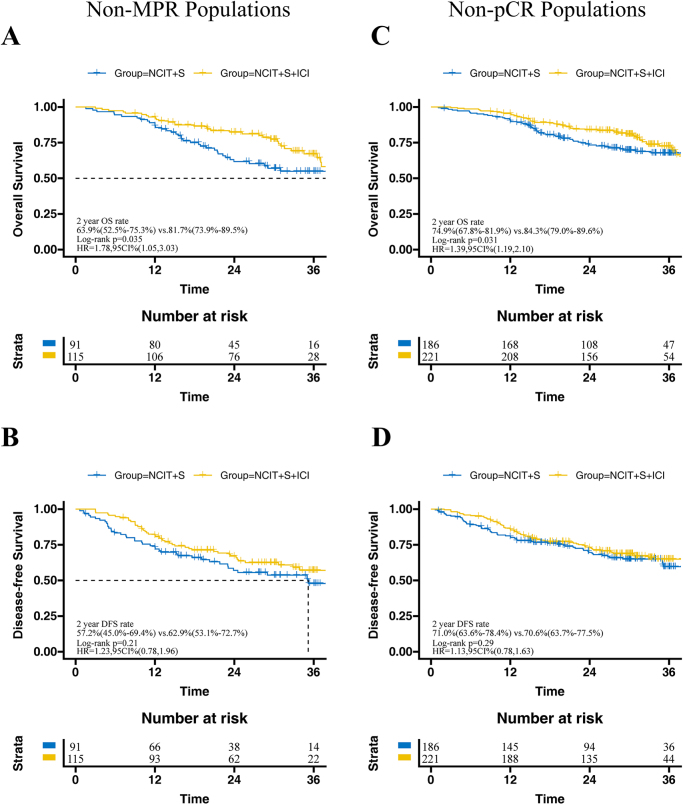
Subgroup analysis of overall survival (OS) and disease-free survival (DFS) were conducted between the NCIT + S and NCIT + S + ICI groups, stratified by Non-MPR (A,B) and Non-pCR (C,D) populations. NCIT, neoadjuvant chemoimmunotherapy; S, surgery; ICI, immune checkpoint inhibitor; MPR, major pathological response; pCR, pathological complete response.

**Figure 4.**
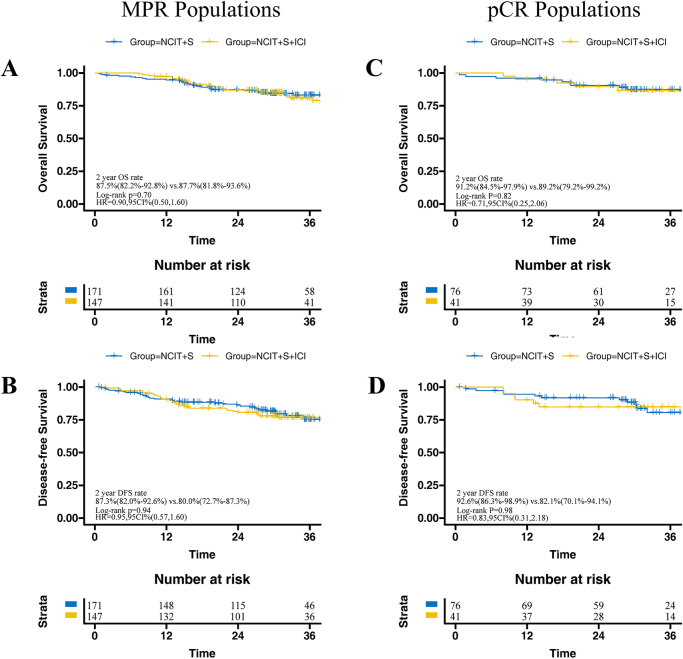
Subgroup analysis of overall survival (OS) and disease-free survival (DFS) were conducted between the NCIT + S and NCIT + S + ICI groups, stratified by MPR (A,B) and pCR (C,D) populations. NCIT, neoadjuvant chemoimmunotherapy; S, surgery; ICI, immune checkpoint inhibitor; MPR, major pathological response; pCR, pathological complete response.

The median follow-up time for survivors in the NCIT + S group was 31.2 months (IQR, 27.9–39.9 months), while the median follow-up time for survivors in the NCIT + S + ICI group was 31.2 months (IQR, 24.0–37.9 months). There was no significant difference in 2-year OS (NCIT + S vs. NCIT + S + ICI: 80.0% vs. 84.5%, HR = 1.15, 95% CI: 0.78–1.70, *P* = 0.12) and DFS (NCIT + S vs. NCIT + S + ICI: 77.7% vs. 72.6%, HR = 0.97, 95% CI: 0.69–1.37, *P* = 0.69) between these two groups in the overall population (Fig. 2). The subgroup analysis based on MPR and pCR status showed that adjuvant immunotherapy provided higher 2-year OS in both Non-MPR patients (NCIT + S + ICI vs. NCIT + S: 81.7% vs. 63.9%, HR = 1.78, 95% CI: 1.05–3.03, *P* = 0.035) and Non-pCR patients (NCIT + S + ICI vs. NCIT + S: 84.3% vs. 74.9%, HR = 1.39, 95% CI: 1.19–2.10, *P* = 0.031), but no significant differences in 2-year DFS (Fig. 3). For the MPR or pCR populations, adjuvant immunotherapy offered no benefits in terms of 2-year OS and DFS (Fig. 4).

Additionally, we conducted subgroup analysis for different TRG status, and the results showed that TRG1, TRG2, and TRG3 had no difference in OS and DFS, but TRG4 population had OS benefit in receiving adjuvant immunotherapy (*P* = 0.002) (Supplemental Digital Content Figure S2, available at, http://links.lww.com/JS9/F201). We also conducted subgroup analysis for different ypN status, and the results showed that there were no statistically significant differences in OS and DFS whether they received adjuvant immunotherapy or not with regard to ypN0 and ypN2 + 3 patients (all *P* > 0.05). For ypN1 patients, adjuvant immunotherapy had a significant benefit in DFS (*P* = 0.02), and there was a significant benefit tendency in OS (*P* = 0.06) (Supplemental Digital Content Figure S3, available at, http://links.lww.com/JS9/F201). Multivariate analysis highlighted ypN + and LVI as independent adverse prognostic indicators for both OS and DFS (Table 2).

**Table 2:** Univariable and multivariable Cox proportional hazard analysis evaluating disease-free survival (DFS) and overall survival (OS)

	DFS				OS			
	Univariate		Multivariate		Univariate		Multivariate	
Variables	*P*	HR (95% CI)	*P*	HR (95% CI)	*P*	HR (95% CI)	*P*	HR (95% CI)
Age								
<60		1.00 (Reference)				1.00 (Reference)		
≥60	0.51	0.88 (0.61–1.27)			0.61	1.12 (0.73–1.71)		<?subhead-end? >
Gender								
Male		1.00 (Reference)				1.00 (Reference)		
Female	0.62	0.90 (0.59–1.37)			0.58	0.87 (0.53–1.42)		<?subhead-end? >
BMI								
< 18		1.00 (Reference)				1.00 (Reference)		1.00 (Reference)
18–24	0.75	1.16 (0.47–2.86)			0.11	0.55 (0.26–1.15)	0.046	0.46 (0.22–0.99)
>18	0.88	1.07 (0.43–2.70)			0.021	0.40 (0.18–0.87)	0.020	0.39 (0.17–0.86)
Smoking history								
No		1.00 (Reference)				1.00 (Reference)		
Yes	0.91	0.98 (0.69–1.40)			0.24	1.29 (0.85–1.96)		<?subhead-end? >
Alcohol abuse								
No		1.00 (Reference)				1.00 (Reference)		
Yes	0.89	1.03 (0.71–1.49)			0.77	1.07 (0.70–1.64)		
Tumor location								
Proximal third		1.00 (Reference)		1.00 (Reference)		1.00 (Reference)		
Middle third	0.007	0.53 (0.33–0.84)	<.001	0.38 (0.23–0.62)	0.22	0.71 (0.41–1.23)		
Distal third	0.13	0.67 (0.39–1.12)	0.004	0.44 (0.25–0.77)	0.58	0.84 (0.45–1.56)		
ECOG-PS								
0		1.00 (Reference)		1.00 (Reference)		1.00 (Reference)		
1	0.016	0.65 (0.46–0.92)	0.008	0.60 (0.42–0.88)	0.87	1.03 (0.70–1.53)		<?subhead-end? >
TRG								
1		1.00 (Reference)		1.00 (Reference)		1.00 (Reference)		1.00 (Reference)
2	0.039	1.75 (1.03–2.97)	0.16	1.90 (0.78–4.63)	0.27	1.39 (0.78–2.49)	0.75	1.16 (0.48–2.82)
3	<.001	3.07 (1.80–5.22)	0.004	3.74 (1.52–9.22)	0.015	2.11 (1.16–3.84)	0.30	1.62 (0.65–4.07)
4	<.001	3.73 (2.19–6.35)	0.006	3.68 (1.45–9.33)	<.001	3.46 (1.98–6.06)	0.20	1.83 (0.73–4.58)
ypN category								
ypN0		1.00 (Reference)		1.00 (Reference)		1.00 (Reference)		1.00 (Reference)
ypN1	0.002	1.93 (1.27–2.95)	<.001	2.28 (1.40–3.73)	0.004	2.07 (1.27–3.38)	0.033	1.87 (1.05–3.34)
ypN2	<.001	2.79 (1.75–4.43)	<.001	2.56 (1.49–4.39)	<.001	3.06 (1.79–5.23)	0.014	2.20 (1.17–4.15)
ypN3	<.001	8.01 (4.42–14.53)	<.001	5.23 (2.44–11.19)	<.001	12.00 (6.54–22.00)	<.001	6.51 (3.06–13.86)
pCR								
No		1.00 (Reference)		1.00 (Reference)		1.00 (Reference)		1.00 (Reference)
Yes	<.001	0.42 (0.25–0.71)	0.23	1.86 (0.68–5.09)	0.007	0.46 (0.26–0.81)	0.67	1.26 (0.44–3.60)
PNI								
Negative		1.00 (Reference)		1.00 (Reference)		1.00 (Reference)		1.00 (Reference)
Positive	<.001	2.58 (1.65–4.02)	0.11	1.51 (0.91–2.51)	<.001	2.99 (1.86–4.79)	0.19	1.44 (0.83–2.51)
LVI								
Negative		1.00 (Reference)		1.00 (Reference)		1.00 (Reference)		1.00 (Reference)
Positive	<.001	2.19 (1.48–3.24)	0.81	0.94 (0.59–1.50)	<.001	3.22 (2.13–4.85)	0.029	1.71 (1.06–2.77)
Adjuvant immunotherapy								
No		1.00 (Reference)				1.00 (Reference)		
Yes	0.87	1.03 (0.73–1.45)			0.48	0.87 (0.59–1.28)		<?subhead-end? >

BMI, body mass index; CI, confidence interval; ECOG-PS, Eastern Cooperative Oncology Group Performance Status; HR, hazard ratio; LVI, lymphatic vessel invasion; pCR, pathological complete response; PNI, perineural invasion; TRG, tumor regression grade.

### Recurrence pattern

For recurrence patterns, the NCIT + S group had similar rates of LR (15.3%) and DM (16.8%), whereas the NCIT + S + ICI group was predominantly characterized by LR (20.2%), with 12.6% of DM. There were no statistically significant differences between the two treatment groups with regard to LR and DM (all *P* > 0.05) (Table 3). We also compared recurrence patterns between the MPR/Non-MPR and pCR/Non-pCR subgroups in both treatment groups. The results showed no differences in either LR or DM within each subgroup (Supplemental Digital Content Table S3, available at, http://links.lww.com/JS9/F202).

**Table 3:** Recurrence patterns of postoperative 2 years between the NCIT + S group and the NCIT + S + ICI group

Variables	Total (*n* = 524)	NCIT + S (*n* = 262)	NCIT + S + ICI (*n* = 262)	*P*
Local recurrence, *n* (%)				0.50
No	431 (82.3)	222 (84.7)	209 (79.8)	
Yes	93 (17.7)	40 (15.3)	53 (20.2)	
Lymph nodes	73 (13.9)	32 (12.2)	41 (15.7)	
Anastomosis	14 (2.7)	6 (2.3)	8 (3.0)	
Anastomosis and lymph nodes	6 (1.1)	2 (0.8)	4 (1.5)	
Distant metastasis, *n* (%)				0.17
No	447 (85.3)	218 (83.2)	229 (87.4)	
Yes	77 (14.7)	44 (16.8)	33 (12.6)	
Bone, *n* (%)				**<0.05**
No	504 (96.2)	246 (93.9)	258 (98.5)	
Yes	20 (3.8)	16 (6.1)	4 (1.5)	
Liver, *n* (%)				0.85
No	495 (94.5)	247 (94.3)	248 (94.7)	
Yes	29 (5.5)	15 (5.7)	14 (5.3)	
Lung, *n* (%)				0.84
No	497 (94.9)	248 (94.7)	249 (95.0)	
Yes	27 (5.1)	14 (5.3)	13 (5.0)	
Brain, *n* (%)				1.00
No	519 (99.1)	259 (98.9)	260 (99.2)	
Yes	5 (0.9)	3 (1.1)	2 (0.8)	
Pleura/pericardium/peritoneum, *n* (%)				0.50
No	515 (98.3)	256 (97.7)	259 (98.9)	
Yes	9 (1.7)	6 (2.3)	3 (1.1)	
Pancreas, *n* (%)				0.25
No	521 (99.4)	259 (98.9)	262 (100.0)	
Yes	3 (0.6)	3 (1.1)	0 (0.0)	
Kidney, *n* (%)				0.25
No	521 (99.4)	259 (98.9)	262 (100.0)	
Yes	3 (0.6)	3 (1.1)	0 (0.0)	
Adrenal gland, *n* (%)				0.48
No	522 (99.6)	260 (99.2)	262 (100.0)	
Yes	2 (0.4)	2 (0.8)	0 (0.0)	
104LN, *n* (%)				0.37
No	519 (99.1)	258 (98.5)	261 (99.6)	
Yes	5 (0.9)	4 (1.5)	1 (0.4)	
Combined, *n* (%)				0.84
No	499 (95.2)	249 (95.0)	250 (95.4)	
Yes	25 (4.8)	13 (5.0)	12 (4.6)	

ICI, immune checkpoint inhibitor; NCIT, neoadjuvant chemoimmunotherapy; S, surgery.

The bold value represent *P* < 0.05.

## Discussion

To the best of our knowledge, the present study is the largest sample multicenter real-world study which investigated the value of adjuvant immunotherapy after NCIT for locally advanced ESCC. The results indicated that adjuvant immunotherapy did not improve 2-year OS or DFS in patients with locally advanced ESCC following NCIT. Nevertheless, patients with residual tumor can derive benefit from adjuvant immunotherapy, which is line with CheckMate 577 trial.

Both surgery and radiotherapy are used locally to treat tumors. In theory, for resectable esophageal cancer, surgery alone can achieve good local tumor control. The addition of systemic therapy, especially neoadjuvant immunotherapy is in order to achieve better systemic tumor control. Several phase 2 clinical trials of NCIT have demonstrated favorable tumor regression with acceptable treatment related toxicity^[10–12]^. Based on these encouraging results, NCIT is now widely used in clinical practice of esophageal cancer in China. Subsequently, the ESCORT-NEO trial as the first phase 3 study worldwide to compare perioperative results of NCIT with neoadjuvant chemotherapy, with NCIT achieving higher pCR (28.0% vs. 4.7%) and MPR (59.1% vs. 20.9%) rates[24]. The other RCT also showed that NCIT had a higher pCR rate (18.6% vs. 4.6%) compared with neoadjuvant chemotherapy[25].

Our previous multicenter, real-world study confirmed that NCIT can improve 2-year OS in patients with locally advanced ESCC compared to NCRT (81.3% vs. 71.3%)[17]. However, patients who underwent NCIT followed by radical esophagectomy still had 18.4% of LR and 13.5% of DM, respectively, during the 2-year postoperative follow-up. In particular, Non-MPR and ypN+ patients were at high risk of recurrence and metastasis, and the prognosis was significantly worse than that of MPR and ypN0 patients[16]. Adjuvant treatments to improve outcomes are clearly needed. Therefore, whether adjuvant immunotherapy after NCIT can effectively reduce recurrence and metastasis and further improve survival needs to be answered urgently. Our results complement CheckMate 577 study, which demonstrated the efficacy of adjuvant nivolumab in Non-pCR ESCC patients after NCRT[17]. However, the neoadjuvant strategy in our study differs significantly, as all patients received NCIT, a regimen that integrates the synergistic effects of chemotherapy and immunotherapy. Emerging evidence suggests that NCIT may induce different anti-tumor immune response with NCRT, potentially altering the tumor microenvironment and immune landscape^[26–28]^. This difference underscores the need for tailored approaches to adjuvant therapy in the context of NCIT. Our findings also provide critical real-world evidence to complement ongoing RCT, such as NCT05043688 and NCT04807673, which aim to evaluate the role of perioperative immunotherapy for locally advanced ESCC^[18,19]^. Until these trials mature, our study offers preliminary but valuable guidance for clinicians managing high-risk ESCC patients.

The pathological results of this study showed that a higher Non-MPR proportion of patients in the NCIT + S + ICI group (43.9% vs. 34.7%) and also higher Non-pCR proportion (84.4% vs. 71.0%) than those in the NCIT + S group. Additionally, patients in the NCIT + S + ICI group had more patients with later pathological stage (ypN +: 50.8% vs. 38.9%; AJCC p-staging III + IV: 51.9% vs. 39.3%). This figure exactly reflects the trend in clinical practice that patients with poor post-NCIT tumor regression prefer to receive adjuvant immunotherapy. In the present study, the NCIT + S group achieved better tumor regression, but the prognosis of Non-MPR or Non-pCR patients in the NCIT + S + ICI group was better. Meanwhile, recurrence rates were similar between NCIT + S and NCIT + S + ICI group, but DM tended to decrease further after adjuvant immunotherapy (16.8% vs. 12.6%). All these findings showed the potential value of adjuvant immunotherapy after NCIT.

Taking DFS as the primary endpoint of the study can compare the differences in recurrence and metastasis between the two groups with or without adjuvant immunotherapy after NCIT within a relatively short period of time. Although OS is the golden standard for survival, obtaining 5-year OS data requires a relatively long period of follow-up. Whether DFS can serve as an alternative indicator for OS remains controversial. However, by analyzing the recurrence and metastasis patterns, it can help us formulate targeted adjuvant treatment plans. CheckMate577 also set DFS as the primary endpoint. It was disclosed at this year’s American Society of Clinical Oncology (ASCO) annual meeting that adjuvant immunotherapy after NCRT did not significantly improve 5-year OS, but the benefit of DFS still persisted.

Due to various biases of retrospective study, there were differences in the 2y-OS between the two groups of patients in the subgroup analysis of Non-MPR and Non-pCR, though there is no significant difference in DFS. The possible explanation was NCIT itself has played a predominant role in reducing postoperative recurrence and metastasis, particularly in the distant metastasis, whereas adjuvant immunotherapy might provide a limited effect[16]. Meanwhile, whether OS can truly benefit still requires a longer follow-up. In the present study, surgical patient stratification for adjuvant immunotherapy was identified via pathological response as a key factor. The significant OS benefit in Non-MPR/Non-pCR patients supports selective escalation of systemic therapy for these high-risk cases. This aligns with the JCOG1109 trial that strengthen neoadjuvant chemotherapy (docetaxel/cisplatin/5-FU) improved OS significantly without increasing surgical morbidity[28]. Conversely, the absence of survival improvement in MPR/pCR patients suggests that routine adjuvant immunotherapy may constitute overtreatment in this low-risk subgroup. Future strategies should combine pathological response evaluation and perioperative biomarkers (e.g., ctDNA dynamics) to guide precision adjuvant immunotherapy[29].

The multivariate analysis showed that ypN+ was an independent risk factor for prognosis after NCIT. Additionally, subgroup analysis of ypN showed that there were no statistically significant differences in OS and DFS in terms of ypN0 and ypN2 + 3 patients whether they received adjuvant immunotherapy or not. However, for ypN1 patients, adjuvant immunotherapy had a significant benefit in DFS (*P* = 0.02), and there was a significant benefit tendency in OS (*P* = 0.06), insufficient sample size or short follow-up may be the reason for the statistical insignificance. The possible explanation was that ypN0 as a group of patients sensitive to NCIT, adjuvant immunotherapy may be redundant. On the other hand, ypN2 + 3 patients as a special group were insensitive to NCIT, and the preoperative lymph node tumor burden was larger, then adjuvant immunotherapy alone cannot significantly improve the prognosis. ypN1 may be the most beneficial group of adjuvant immunotherapy. There were no statistically significant differences in OS and DFS with regard to TRG1, TRG2, and TRG3 patients, but TRG4 population had OS benefit in receiving adjuvant immunotherapy (*P* = 0.002). This law is line with the 8th edition AJCC-ypTNM staging of esophageal cancer[21]. After receiving neoadjuvant therapy, the status of ypN has a greater impact on prognosis than that of ypT^[20,30,31]^.

Despite the strengths of our study, several limitations should be acknowledged. First, as a retrospective study, potential biases inherent to observational data such as clinical decisions related to adjuvant therapy administration or the presence of postoperative complications cannot be fully excluded, although rigorous PSM was employed to mitigate confounding factors. Second, the heterogeneity in NCIT regimens and adjuvant immunotherapy agents and the number of completed cycles across participating centers may have introduced variability in treatment outcomes. Third, the relatively short follow-up period precludes definitive conclusions about long-term survival benefits. Fourth, the safety evaluation of adjuvant immunotherapy is missing due to the data of immune-related adverse effects were not accurately recorded. Lastly, the lack of robust biomarker analysis (such as PD-L1, ctDNA) limits our ability to identify predictive factors for adjuvant immunotherapy efficacy. Future prospective studies should aim to validate our findings in larger, more diverse populations and explore the integration of biomarkers such as ctDNA to refine patient selection. Additionally, mechanistic studies investigating the interplay between residual tumor burden and immune response will be critical to optimizing adjuvant immunotherapy strategies.

## Conclusion

In patients with locally advanced ESCC who undergo NCIT and radical surgery, adjuvant immunotherapy did not result in a significant survival benefit for the overall cohort. However, patients with residual tumor after NCIT can derive survival benefit from adjuvant immunotherapy, which is line with CheckMate 577 trial. Further investigation is needed to identify the optimal candidates for this treatment strategy.

## Supplementary Material

## Data Availability

De-identified individual participant data, along with the study protocol, statistical analysis plan, and informed consent form, will be made available upon reasonable request to the corresponding author, following publication. Data will be available for a period of 6 months following publication, for the purposes of academic research only, and after the approval of a methodologically sound research proposal.
