# Supplementary material for: Adjuvant immunotherapy for esophageal squamous cell carcinoma after neoadjuvant chemoimmunotherapy: a multicenter real-world study
Source: Int J Surg. 2025 Sep 24;112(1):1284–94. doi: 10.1097/JS9.0000000000003546 (PMC12825888; doi:10.1097/JS9.0000000000003546)
Supplement: Supplementary file 1 [file js9-112-1284-001.docx]

**Supplemental Figure 1:** A scatter plot of the absolute mean differences in baseline data between the NCIT+S and NCIT+S+ICI groups before and after matching

Red circles: before matching; Blue triangles: after matching


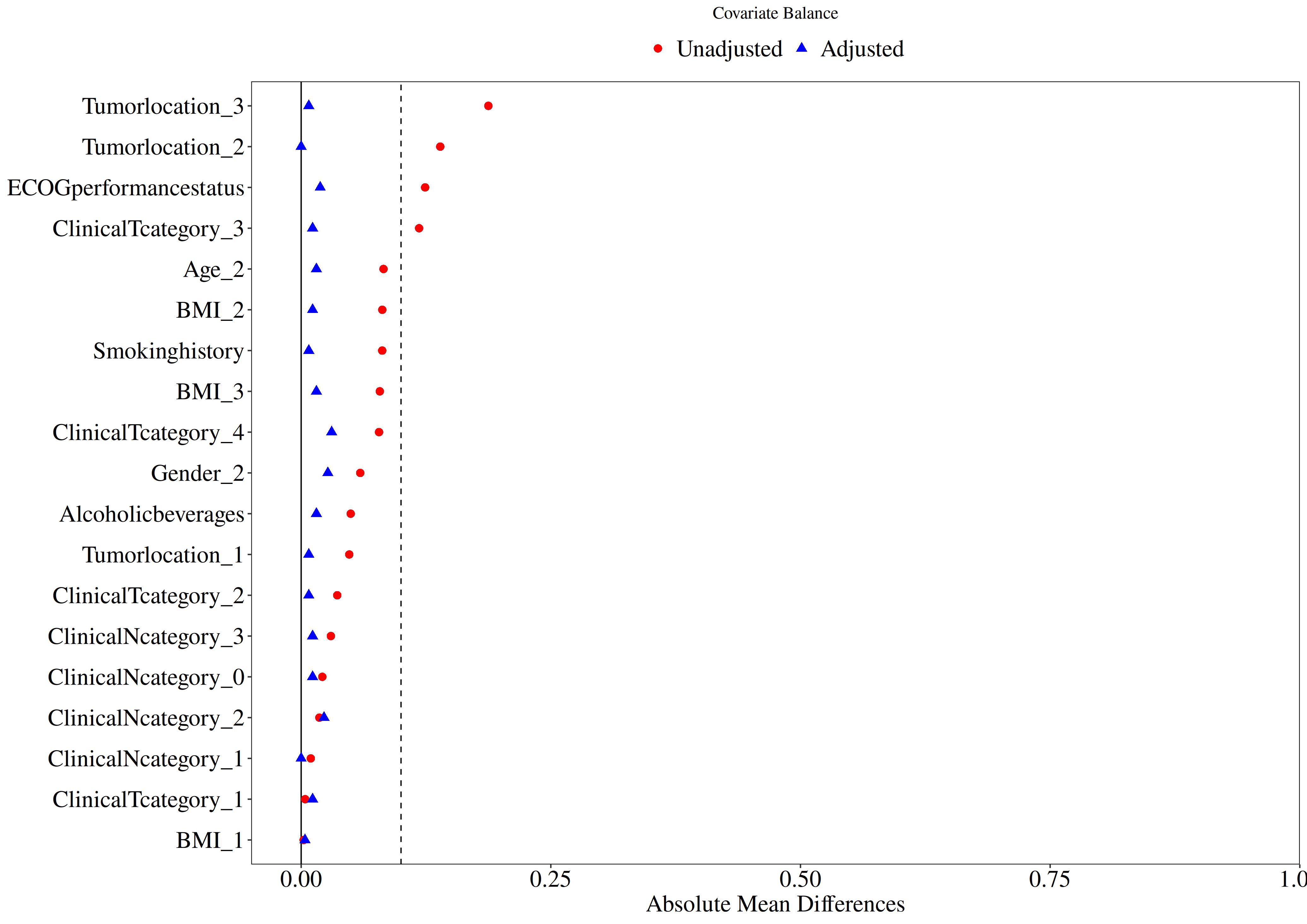
**Supplemental Figure 2:** Subgroup analysis of overall survival (A) and disease-free survival (B) were conducted between the NCIT+S and NCIT+S+ICI groups, stratified by TRG category.

**

**

**Supplemental Figure 3:** Subgroup analysis of overall survival (A) and disease-free survival (B) were conducted between the NCIT+S and NCIT+S+ICI groups, stratified by ypN category.
