# Supplementary material for: Adjuvant immunotherapy for esophageal squamous cell carcinoma after neoadjuvant chemoimmunotherapy: a multicenter real-world study
Source: Int J Surg. 2025 Sep 24;112(1):1284–94. doi: 10.1097/JS9.0000000000003546 (PMC12825888; doi:10.1097/JS9.0000000000003546)
Supplement: Supplementary file 2 [file js9-112-1284-002.docx]

Supplemental Table 1: Pathological outcomes of patients between the NCIT+S group and the NCIT+S+ICI group

| Variables | Total (n = 524) | NCIT+S  (n = 262) | NCIT+S+ICI  (n = 262) | *P* |
| --- | --- | --- | --- | --- |
|  |  |  |  |  |
| yPT category, n(%) |  |  |  | 0.14 |
| ypT0 | 167 (31.9) | 97 (37.0) | 70 (26.7) |  |
| ypT1 | 104 (19.8) | 46 (17.6) | 58 (22.1) |  |
| ypT2 | 74 (14.1) | 33 (12.6) | 41 (15.7) |  |
| ypT3 | 163 (31.1) | 79 (30.1) | 84 (32.1) |  |
| ypT4 | 16 (3.1) | 7 (2.7) | 9 (3.4) |  |
| ypN category, n(%) |  |  |  | **0.045** |
| ypN0 | 289 (55.2) | 160 (61.1) | 129 (49.2) |  |
| ypN1 | 142 (27.1) | 64 (24.4) | 78 (29.8) |  |
| ypN2 | 71 (13.5) | 28 (10.7) | 43 (16.4) |  |
| ypN3 | 22 (4.2) | 10 (3.8) | 12 (4.6) |  |
| TRG, n(%) |  |  |  | **0.046** |
| 1 | 167 (31.9) | 97 (37.0) | 70 (26.7) |  |
| 2 | 156 (29.8) | 78 (29.8) | 78 (29.8) |  |
| 3 | 97 (18.5) | 42 (16.0) | 55 (21.0) |  |
| 4 | 104 (19.8) | 45 (17.2) | 59 (22.5) |  |
| MPR, n(%) |  |  |  | **0.032** |
| No | 206 (39.3) | 91 (34.7) | 115 (43.9) |  |
| Yes | 318 (60.7) | 171 (65.3) | 147 (56.1) |  |
| pCR, n(%) |  |  |  | **<.001** |
| No | 407 (77.7) | 186 (71.0) | 221 (84.4) |  |
| Yes | 117 (22.3) | 76 (29.0) | 41 (15.6) |  |
| AJCC p-staging, n(%) |  |  |  | **0.004** |
| Ⅰ+Ⅱ | 285 (54.4) | 159 (60.7) | 126 (48.1) |  |
| Ⅲ+Ⅳ | 239 (45.6) | 103 (39.3) | 136 (51.9) |  |
| PNI, n(%) |  |  |  | 0.55 |
| No | 474 (90.5) | 235 (89.7) | 239 (91.2) |  |
| Yes | 50 (9.5) | 27 (10.3) | 23 (8.8) |  |
| LVI, n(%) |  |  |  | 0.30 |
| No | 435 (83.0) | 222 (84.7) | 213 (81.3) |  |
| Yes | 89 (14.0) | 40 (15.3) | 49 (18.7) |  |
| TotalLND, M (Q₁, Q₃) | 28.0 (20.0, 37.0) | 29.0 (21.0, 36.0) | 26.0 (19.0, 38.0) | 0.14 |
| CervicalLND, M (Q₁, Q₃) | 3.0 (0.0, 11.0) | 4.0 (0.0, 12.0) | 2.0 (0.0, 11.0) | 0.08 |
| ThoracicLND, M (Q₁, Q₃) | 13.0 (8.0, 20.0) | 14.0 (9.0, 20.0) | 13.0 (8.0, 20.0) | 0.69 |
| AbdominalLND, M (Q₁, Q₃) | 8.0 (0.0, 13.0) | 8.0 (0.0, 14.0) | 7.0 (0.0, 12.0) | 0.23 |
| 106recR, M (Q₁, Q₃) | 2.0 (1.0, 4.0) | 2.0 (1.0, 4.0) | 2.0 (1.0, 4.0) | 0.40 |
| 106recL, M (Q₁, Q₃) | 2.0 (1.0, 3.0) | 2.0 (1.0, 3.0) | 2.0 (1.0, 3.0) | 0.55 |

Supplemental Table 2: Postoperative complications between the NCIT+S group and the NCIT+S+ICI group

| Variables | Total (n = 524) | NCIT+S (n = 262) | NCIT+S+ICI (n = 262) | *P* |
| --- | --- | --- | --- | --- |
|  |  |  |  |  |
| Overall complication |  |  |  | 0.13 |
| No | 293 (55.9) | 155 (59.2) | 138 (52.7) |  |
| Yes | 231 (44.1) | 107 (40.8) | 124 (47.3) |  |
| Respiratory complication, n(%) |  |  |  | 0.09 |
| No | 358 (68.3) | 170 (64.9) | 188 (71.8) |  |
| Yes | 166 (31.7) | 92 (35.1) | 74 (28.2) |  |
| Pneumonia, n(%) |  |  |  | 0.05 |
| No | 388 (74.1) | 184 (70.2) | 204 (77.9) |  |
| Yes | 136 (25.9) | 78 (29.8) | 58 (22.1) |  |
| Respiratory failure, n(%) |  |  |  | 0.43 |
| No | 509 (97.1) | 253 (96.6) | 256 (97.7) |  |
| Yes | 15 (2.9) | 9 (3.4) | 6 (2.3) |  |
| ARDS, n(%) |  |  |  | 1.00 |
| No | 517 (98.7) | 258 (98.5) | 259 (98.9) |  |
| Yes | 7 (1.3) | 4 (1.5) | 3 (1.1) |  |
| The effusion requires treatment, n(%) |  |  |  | 0.09 |
| No | 393 (75.0) | 188 (71.8) | 205 (78.2) |  |
| Yes | 131 (25.0) | 74 (28.2) | 57 (21.8) |  |
| Pneumothorax requires treatment, n(%) |  |  |  | 0.24 |
| No | 512 (97.7) | 258 (98.5) | 254 (97.0) |  |
| Yes | 12 (2.3) | 4 (1.5) | 8 (3.0) |  |
| Cardiac complication, n(%) |  |  |  | 0.52 |
| No | 501 (95.6) | 249 (95.0) | 252 (96.2) |  |
| Yes | 23 (4.4) | 13 (5.0) | 10 (3.8) |  |
| Anastomotic leak, n(%) |  |  |  | 0.88 |
| No | 471 (89.9) | 235 (89.7) | 236 (90.1) |  |
| Yes | 53 (10.1) | 27 (10.3) | 26 (9.9) |  |
| Recurrent laryngeal nerve palsy, n(%) |  |  |  | 0.27 |
| No | 445 (84.9) | 218 (83.2) | 227 (86.6) |  |
| Yes | 79 (15.1) | 44 (16.8) | 35 (13.4) |  |
| Chylothorax, n(%) |  |  |  | 0.40 |
| 0 | 511 (97.5) | 257 (98.1) | 254 (97.0) |  |
| 1 | 13 (2.5) | 5 (1.9) | 8 (3.0) |  |
| Re-operation, n(%) |  |  |  | 1.00 |
| No | 522 (99.6) | 261 (99.6) | 261 (99.6) |  |
| Yes | 2 (0.4) | 1 (0.4) | 1 (0.4) |  |
| 90-d hospital mortality, n(%) |  |  |  | 1.00 |
| No | 523 (99.8) | 261 (99.6) | 262 (100.0) |  |
| Yes | 1 (0.2) | 1 (0.4) | 0 (0.0) |  |

Supplemental Table 3: Recurrence patterns of MPR/Non-MPR and pCR/Non-pCR between the NCIT+S group and NCIT+S+ICI group.

|  | MPR | |  |  | Non-MPR | |  |  | pCR | |  | Non-pCR | |  |  |
| --- | --- | --- | --- | --- | --- | --- | --- | --- | --- | --- | --- | --- | --- | --- | --- |
|  | NCIT+S  (n = 171) | NCIT+S+ICI  (n = 147) | *P* |  | NCIT+S  (n = 91) | NCIT+S+ICI  (n = 115) | *P* |  | NCIT+S  (n = 76) | NCIT+S+ICI  (n = 41) | *P* | NCIT+S  (n = 186) | NCIT+S+ICI  (n = 221) | *P* |  |
| Localregional recurrence, n(%) |  |  | 0.67 |  |  |  | 0.93 |  |  |  | 0.64 |  |  | 0.84 |  |
| None | 152 (88.9) | 125 (85.0) |  |  | 70 (76.9) | 84 (73.1) |  |  | 71 (93.4) | 37 (90.2) |  | 151 (81.2) | 172 (77.8) |  |  |
| Lymph nodes | 15 (8.8) | 18 (12.2) |  |  | 17 (18.7) | 23 (20.0) |  |  | 4 (5.3) | 4 (9.8) |  | 28 (15.0) | 37 (16.8) |  |  |
| Anastomosis | 3 (1.7) | 2 (1.4) |  |  | 3 (3.3) | 6 (5.2) |  |  | 1 (1.3) | 0 (0.0) |  | 5 (2.7) | 8 (3.6) |  |  |
| Anastomosis and lymph nodes | 1 (0.6) | 2 (1.4) |  |  | 1 (1.1) | 2 (1.7) |  |  | 0 (0.0) | 0 (0.0) |  | 2 (1.1) | 4 (1.8) |  |  |
| Distant metastasis, n(%) |  |  | 0.41 |  |  |  | 0.12 |  |  |  | 0.32 |  |  | 0.15 |  |
| No | 151 (88.3) | 134 (91.2) |  |  | 67 (73.63) | 95 (82.61) |  |  | 69 (90.8) | 40 (97.6) |  | 149 (80.1) | 189 (85.5) |  |  |
| Yes | 20 (11.7) | 13 (8.8) |  |  | 24 (26.37) | 20 (17.39) |  |  | 7 (9.2) | 1 (2.4) |  | 37 (19.9) | 32 (14.5) |  |  |
| Bone, n(%) |  |  | 0.39 |  |  |  | **<.01** |  |  |  | 0.54 |  |  | **<.01** |  |
| No | 165 (96.5) | 145 (98.6) |  |  | 81 (89.0) | 113 (98.3) |  |  | 74 (97.4) | 41 (100.0) |  | 172 (92.5) | 217 (98.2) |  |  |
| Yes | 6 (3.5) | 2 (1.4) |  |  | 10 (11.0) | 2 (1.7) |  |  | 2 (2.6) | 0 (0.0) |  | 14 (7.5) | 4 (1.8) |  |  |
| Liver, n(%) |  |  | 0.24 |  |  |  | 0.13 |  |  |  | **#** |  |  | 0.50 |  |
| No | 167 (97.7) | 140 (95.2) |  |  | 80 (87.9) | 108 (93.9) |  |  | 76 (100.0) | 41 (100.0) |  | 171 (91.9) | 207 (93.7) |  |  |
| Yes | 4 (2.3) | 7 (4.8) |  |  | 11 (12.1) | 7 (6.1) |  |  | 0 (0.0) | 0 (0.0) |  | 15 (8.1) | 14 (6.3) |  |  |
| Lung, n(%) |  |  | 0.57 |  |  |  | 0.31 |  |  |  | 1.00 |  |  | 0.66 |  |
| No | 165 (96.5) | 140 (95.2) |  |  | 83 (91.2) | 109 (94.8) |  |  | 74 (97.4) | 40 (97.6) |  | 174 (93.6) | 209 (94.6) |  |  |
| Yes | 6 (3.5) | 7 (4.8) |  |  | 8 (8.8) | 6 (5.2) |  |  | 2 (2.6) | 1 (2.4) |  | 12 (6.4) | 12 (5.4) |  |  |
| Pleura/Pericardium/Perioneum, n(%) |  |  | 1.00 |  |  |  | 1.00 |  |  |  | **#** |  |  | 0.85 |  |
| No | 170 (99.4) | 147 (100.0) |  |  | 89 (97.8) | 113 (98.3) |  |  | 76 (100.0) | 41 (100.0) |  | 183 (98.4) | 219 (99.1) |  |  |
| Yes | 1 (0.6) | 0 (0.0) |  |  | 2 (2.2) | 2 (1.7) |  |  | 0 (0.0) | 0 (0.0) |  | 3 (1.6) | 2 (0.9) |  |  |
| Brain, n(%) |  |  | 0.17 |  |  |  | 1.00 |  |  |  | 0.50 |  |  | 1.00 |  |
| No | 167 (97.7) | 147 (100.0) |  |  | 89 (97.8) | 112 (97.4) |  |  | 73 (96.1) | 41 (100.0) |  | 183 (98.4) | 218 (98.6) |  |  |
| Yes | 4 (2.3) | 0 (0.0) |  |  | 2 (2.2) | 3 (2.6) |  |  | 3 (3.9) | 0 (0.0) |  | 3 (1.6) | 3 (1.4) |  |  |
| Pancreas, n(%) |  |  | 1.00 |  |  |  | 0.19 |  |  |  | 1.00 |  |  | 0.21 |  |
| No | 170 (99.4) | 147 (100.0) |  |  | 89 (97.8) | 115 (100.0) |  |  | 75 (98.7) | 41 (100.0) |  | 184 (98.9) | 221 (100.0) |  |  |
| Yes | 1 (0.6) | 0 (0.0) |  |  | 2 (2.2) | 0 (0.0) |  |  | 1 (1.3) | 0 (0.0) |  | 2 (1.1) | 0 (0.0) |  |  |
| Kidney, n(%) |  |  | 1.00 |  |  |  | 0.19 |  |  |  | **#** |  |  | 0.19 |  |
| No | 170 (99.4) | 147 (100.0) |  |  | 89 (97.8) | 115 (100.0) |  |  | 76 (100.0) | 41 (100.0) |  | 183 (98.4) | 221 (100.0) |  |  |
| Yes | 1 (0.6) | 0 (0.0) |  |  | 2 (2.2) | 0 (0.0) |  |  | 0 (0.0) | 0 (0.0) |  | 3 (1.6) | 0 (0.0) |  |  |
| Adrenal gland, n(%) |  |  | 1.00 |  |  |  | 0.44 |  |  |  | **#** |  |  | 0.21 |  |
| No | 170 (99.4) | 147 (100.0) |  |  | 90 (98.9) | 115 (100.0) |  |  | 76 (100.0) | 41 (100.00) |  | 184 (98.9) | 221 (100.0) |  |  |
| Yes | 1 (0.6) | 0 (0.0) |  |  | 1 (1.1) | 0 (0.0) |  |  | 0 (0.0) | 0 (0.00) |  | 2 (1.1) | 0 (0.0) |  |  |
| 104LN, n(%) |  |  | 1.00 |  |  |  | 0.46 |  |  |  | **#** |  |  | 0.27 |  |
| No | 170 (99.4) | 147 (100.0) |  |  | 88 (96.7) | 114 (99.1) |  |  | 76 (100.0) | 41 (100.00) |  | 182 (97.9) | 220 (99.6) |  |  |
| Yes | 1 (0.6) | 0 (0.0) |  |  | 3 (3.3) | 1 (0.9) |  |  | 0 (0.0) | 0 (0.00) |  | 4 (2.1) | 1 (0.4) |  |  |
| Combined |  |  | 0.96 |  |  |  | 0.65 |  |  |  | 1.00 |  |  | 0.52 |  |
| No | 165 (96.5) | 142 (96.6) |  |  | 84 (92.3) | 108 (93.9) |  |  | 75 (98.7) | 40 (97.6) |  | 174 (93.6) | 210 (95.0) |  |  |
| Yes | 6 (3.5) | 5 (3.4) |  |  | 7 (7.7) | 7 (6.1) |  |  | 1 (1.3) | 1 (2.4) |  | 12 (6.4) | 11 (5.0) |  |  |
|  |  |  |  |  |  |  |  |  |  |  |  |  |  |  |  |
